# The causality between obstructive sleep apnea and ventricular structure and function: a bidirectional Mendelian randomization study

**DOI:** 10.3389/fgene.2023.1266869

**Published:** 2023-10-10

**Authors:** Wanli Sun, Fan Yang, Yiyuan Yang, Xin Su, Yanwei Xing

**Affiliations:** Guang’anmen Hospital, China Academy of Chinese Medical Sciences, Beijing, China

**Keywords:** obstructive sleep apnea, left ventricular, right ventricular, genetic association, Mendelian randomization

## Abstract

**Background:** Multiple observational studies have discovered a substantial link between obstructive sleep apnea (OSA) and ventricular dysfunction. However, conventional observational studies are vulnerable to causal reversal and confounding, making it challenging to infer the causes of effects and their direction.

**Methods:** With the help of a bidirectional, two-sample Mendelian randomization (MR) study, we assessed the potential causality between OSA and left and right ventricular (LV, RV) structure and function. We conducted our analysis utilizing summary data from genome-wide association studies of OSA (16,761 cases and 201,194 controls) in the FinnGen Study, as well as LV (36,041 participants) and RV (29,506 participants) in the UK Biobank cardiovascular magnetic resonance research. The inverse variance weighted (IVW) was selected as the main strategy, with the MR-Egger and weighted median methods serving as supplements. Other methods were employed as sensitivity analysis tools to look at heterogeneity and pleiotropy, including MR-Egger intercept, Cochran Q statistic, MR-PRESSO, and leave-one-out analysis.

**Results:** In the primary IVW analysis, genetically predicted OSA was strongly causative on LV end-diastolic volume (*β* = 0.114, 95% CI = 0.034–0.194, *p* = 0.006) and LV stroke volume (*β* = 0.111, 95% CI = 0.031–0.191, *p* = 0.007), and genetically predicted LV ejection fraction was linked to an increased risk of OSA (OR = 1.161, 95% CI = 1.029–1.309, *p* = 0.015). However, there was no connection found between OSA and any RV parameters.

**Conclusion:** Our genetic analysis raises a potential causative link between OSA and ventricular structure and function, which may improve the knowledge of OSA as a risk factor for cardiovascular disease by demonstrating a direct impact on cardiac structure and function.

## 1 Introduction

Obstructive sleep apnea (OSA) is a widespread sleep disease that may affect up to one billion individuals globally (Benjafield et al., 2019). OSA is characterized by intermittent upper airway obstruction with hypoxia, increased ventilation and sleep disruption. Several studies have shown that OSA promotes sympathetic activity, oxidative stress, and inflammation, resulting in adverse cardiac structures and an increased risk of cardiovascular outcomes (Dempsey et al., 2010). For example, the prevalence of OSA can range from 40% to 80% in individuals with hypertension, heart failure (HF), coronary artery disease (CAD), pulmonary hypertension, atrial fibrillation (AF), and stroke (Marin et al., 2005; Yaggi et al., 2005; Gottlieb et al., 2010; Gami et al., 2013; Yeghiazarians et al., 2021). Furthermore, earlier research has indicated that reduced left ventricular (LV) systolic function and right ventricular (RV) function are associated with lower nocturnal oxygen saturation owing to OSA (Korcarz et al., 2016). LV filling pressures, LV mass, RV size, and RV pressures were significantly reduced in patients with severe OSA after 3 months of continuous positive airway pressure (CPAP) therapy (Shivalkar et al., 2006; Bakker et al., 2020). However, as the information currently available about the link between OSA and ventricular dysfunction is based on traditional observational research, remaining confounding and reverse causality cannot be completely ruled out. Furthermore, cardiovascular magnetic resonance (CMR) imaging is the gold standard for evaluating LV/RV function with more excellent image quality and reproducibility; nevertheless, most studies using conventional echocardiography to determine the association between OSA and cardiac function are somewhat constrained. Therefore, the causality between OSA and ventricular structure and function is still uncertain.

The Mendelian randomization (MR) strategy can be employed to establish if an exposure has a causal connection to the onset of a disease (Katan, 1986; Lawlor et al., 2008; Emdin et al., 2017). Given that genetic variations are already distributed at random depending on parental genetics, the MR method can eliminate the confounding influence of the external environment. In order to assess the causality between exposures and desired outcomes, single nucleotide polymorphisms (SNPs) may thus be utilized as instrumental variables (IVs). Furthermore, extensive open-source genome-wide association studies (GWAS) summary statistics offer the appropriate datasets for accurately and affordably investigating the bidirectional causal effect. Hence, this study implemented genetic instruments to carry out a bidirectional MR study to examine the causality between OSA and LV/RV structure and function captured by CMR.

## 2 Materials and methods

### 2.1 Study design

**FIGURE 1:**
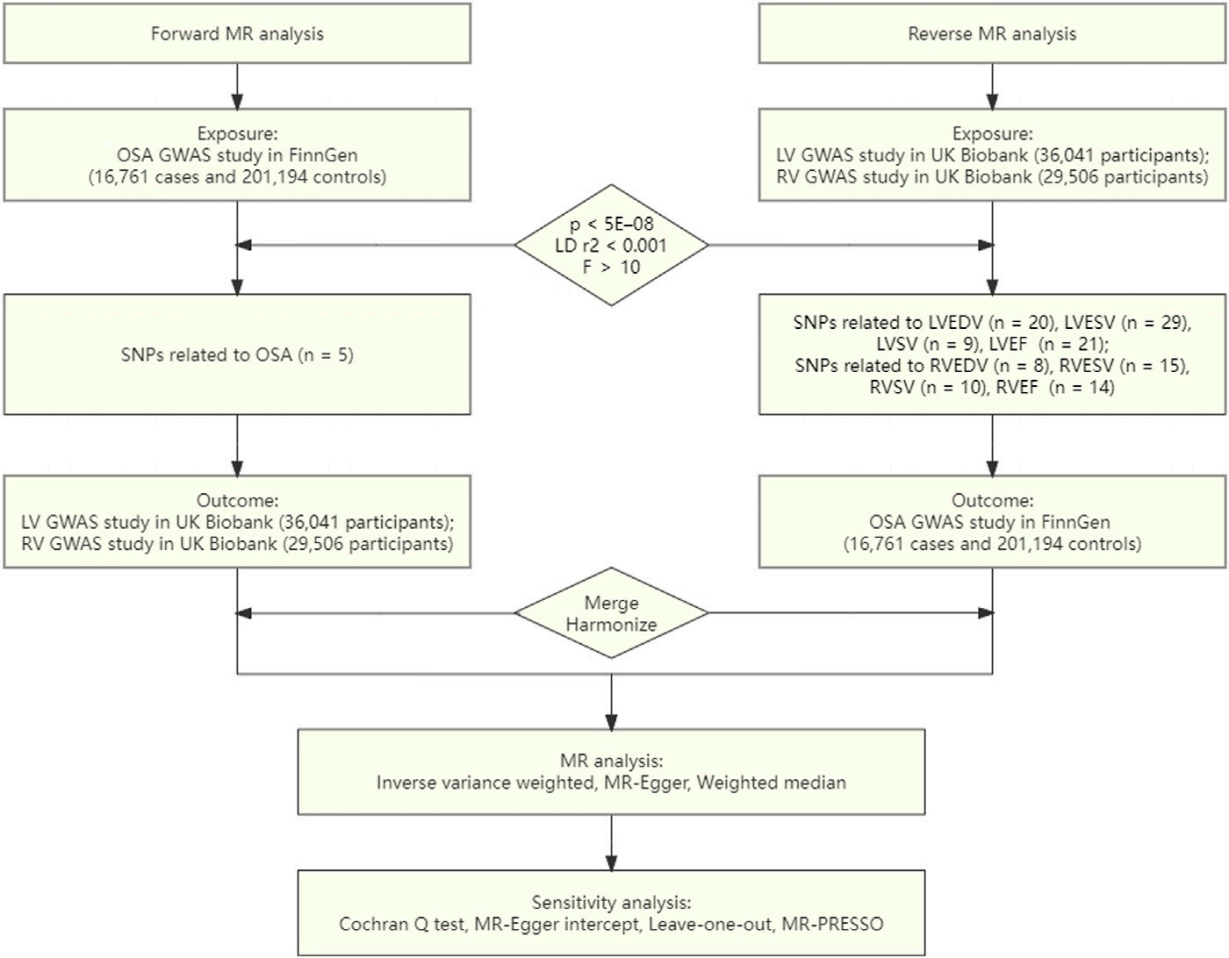
Flowchart of the causal inference between OSA and LV/RV parameters.

Three fundamental hypotheses must be met by the genetic variations utilized for the IVs in the MR study (Benjafield et al., 2019): There is a substantial correlation between the exposure and the IVs (Dempsey et al., 2010). Any confounding factors have no impact on the IVs (Gami et al., 2013). Merely the investigated exposure, not through other pathways, are the IVs linked to the outcomes (Emdin et al., 2017). The objective of the current investigation was to explore the causative inference between OSA and ventricular structure and function via conducting a univariate bidirectional two-sample MR analysis. First, we evaluated whether OSA was causally related to LV/RV structure and function. In the second stage, we also examined whether genetically determined LV/RV structure and function were causally related to OSA. An overview of the study design is presented in Figure 1.

### 2.2 Data sources

We used publicly accessible GWAS summary-level data for our analysis. From a recently published GWAS, containing 16,761 patients and 201,194 controls in the FinnGen Study, we extracted the genetic correlations of the IVs with OSA (Strausz et al., 2021). Using hospital biobank samples, prospective and retrospective epidemiological cohorts, and disease-based cohorts, FinnGen is a significant biobank study with the goal of genotyping 500,000 Finns. Subjective symptoms, a clinical examination, and sleep registration with an apnoea-hypopnoea index of five per hour or a respiratory event index of five per hour are utilized to diagnose OSA, which is defined by the International Classification of Diseases (ICD) codes (ICD10: G47.3, ICD9: 3472A).

We used summary statistics from the UK Biobank, a population-based cohort with more than 500,000 subjects, for the research of the LV/RV structure and function (Petersen et al., 2016; Bycroft et al., 2018). A sizable imaging substudy for this cohort had intentions to conduct CMR on 100,000 individuals. With electrocardiographic gating for cardiac synchronization, CMR was carried out using 1.5-T scanners (MAGNETOM Aera, Syngo Platform vD13A, Siemens Healthcare) (Petersen et al., 2016). Due to its higher image quality and reproducibility, CMR imaging is thought to be the gold standard for thorough heart examination (Backhaus et al., 2019). The LV end-diastolic volume (LVEDV), LV end-systolic volume (LVESV), LV stroke volume (LVSV), and LV ejection fraction (LVEF) imaging phenotypes, which are clinically relevant and prognostically significant, were the focus of this investigation. A total of 36,041 UK Biobank individuals participated in the GWAS summary statistics of LV parameters; none of them had been previously diagnosed with congestive HF, CAD, or dilated cardiomyopathy at the time of registration (Pirruccello et al., 2020). The individuals were mostly female (52.9%) and had an average age of 64 at the time of the CMR. LVEDV, LVESV, LVSV, and LVEF had respective mean values of 140 mL, 49 mL, 88 mL, and 65%. The RV GWAS study, which included 29,506 Europeans without a history of myocardial infarction or HF, examined four RV indicators [RV end-diastolic volume (RVEDV), RV end-systolic volume (RVESV), RV stroke volume (RVSV), and RV ejection fraction (RVEF)] (Aung et al., 2022). The cohort was 47% male and had an average age of 63 during the imaging visit. 157mL, 68mL, 89mL, and 57%, respectively, were the average values for RVEDV, RVESV, RVSV, and RVEF.

### 2.3 Genetic instrument selection

We performed strict SNPs filtration for forward analysis using OSA as the exposure. In order to meet the requirements of MR, we first chose independent SNPs that were significantly linked with OSA at the threshold of genome-wide significance [p < 5E–08, linkage disequilibrium (LD) r^2^ < 0.001, within a 10,000 kb distance]. To further evaluate the potency of the chosen IVs, the *R* ^2^ and F statistics of the SNPs were estimated applying the equation below: *R* ^2^ = 2 × EAF × (1-EAF) × β^2^ and the F statistic = *R* ^2^×(N−2)/(1−*R* ^2^), where EAF stands for effect allele frequency, *β* for an estimate of the genetic impact of each SNP upon exposure GWAS data, and N for a sample size of the exposure GWAS data. The SNP is powerful enough to counteract any possible bias if the F value is higher than 10 (Pierce et al., 2011).

From the outcome data, we then extracted the exposure-SNPs. The effect direction was harmonized to guarantee that the effect of exposure-SNPs and the outcome-SNPs was based on the same allele. We substituted instruments with proxy SNPs (LD r^2^ > 0.8) for the SNPs that were not present in the outcome data by looking up the SNPs online at http://snipa.helmholtz-muenchen.de/snipa3/. We also eliminated SNPs containing palindrome alleles (A/T or G/C) to avoid problems with strand ambiguity. Similar to forward analysis, reverse analysis employed the same genetic instrument selection procedures. Because just few significant RVSV SNPs were discovered using the P < 5E–08 criteria, SNPs were designated as RVSV IVs at P < 5E–07 in order to ensure adequate IVs and avoid weak instrument bias.

### 2.4 MR analysis

The major analysis employed in both the forward and reverse MR studies was the inverse variance weighted (IVW) method, which looked at the causal connection between OSA and LV/RV. The IVW approach, which posits that all SNPs can impact the result exclusively via relevance exposure without directional pleiotropy, is thought to be the most reliable MR method. To augment IVW estimations, the MR-Egger and weighted median (WM) approaches were utilized since they could offer more reliable estimates in a wider variety of scenarios, however, at the cost of diminished statistical power. Even with up to 50% of the genetic variance being invalid, the results using the MR-Egger yielded reliable findings (Bowden et al., 2015). When the share of weights from invalid variations was less than 50%, the WM method provided effect estimates (Bowden et al., 2016). The robustness of the causal inference was improved by the direction and magnitude being consistent across the three approaches.

We also performed sensitivity analysis with several methods to analyze the heterogeneities and pleiotropy of IVs (Burgess et al., 2017). The Cochran Q test was first used to measure heterogeneity. Furthermore, any potential directional pleiotropy was determined employing the intercept from the MR-Egger regression. Additionally, via identifying and eliminating possibly pleiotropic outliers, the MR pleiotropy residual sum and outlier (MR-PRESSO) approach provides a corrective test (Verbanck et al., 2018). Finally, the causative influence of outlying IVs was evaluated via leave-one-out analysis, which gradually removed each IV from the MR analysis. In our study, we performed Bonferroni correction according to the classification of LV/RV structures (LV/RV: 0.05/4 = 0.0125). Results for causal effects were considered statistically significant when *p* < 0.0125 (Bonferroni correction) to represent strong evidence of a causal association. Associations with *p* values below 0.05 but above 0.0125 were considered suggestive evidence of association. All analyses were conducted in the R software (version 4.0.2) with R packages “TwosampleMR” (version 0.5.6) and “MR-PRESSO” (version 1.0) (Hemani et al., 2018; Verbanck et al., 2018).

## 3 Results

### 3.1 Causal effects of OSA on LV/RV

Five SNPs linked to OSA were utilized for forward MR analysis after rigorous instrument selection. There were no weak instruments included in our analysis, according to the F statistics of the chosen IVs, which ranged from 504 to 1117. Supplementary Table S1 presents detailed information on the considered variations of OSA.

**FIGURE 2:**
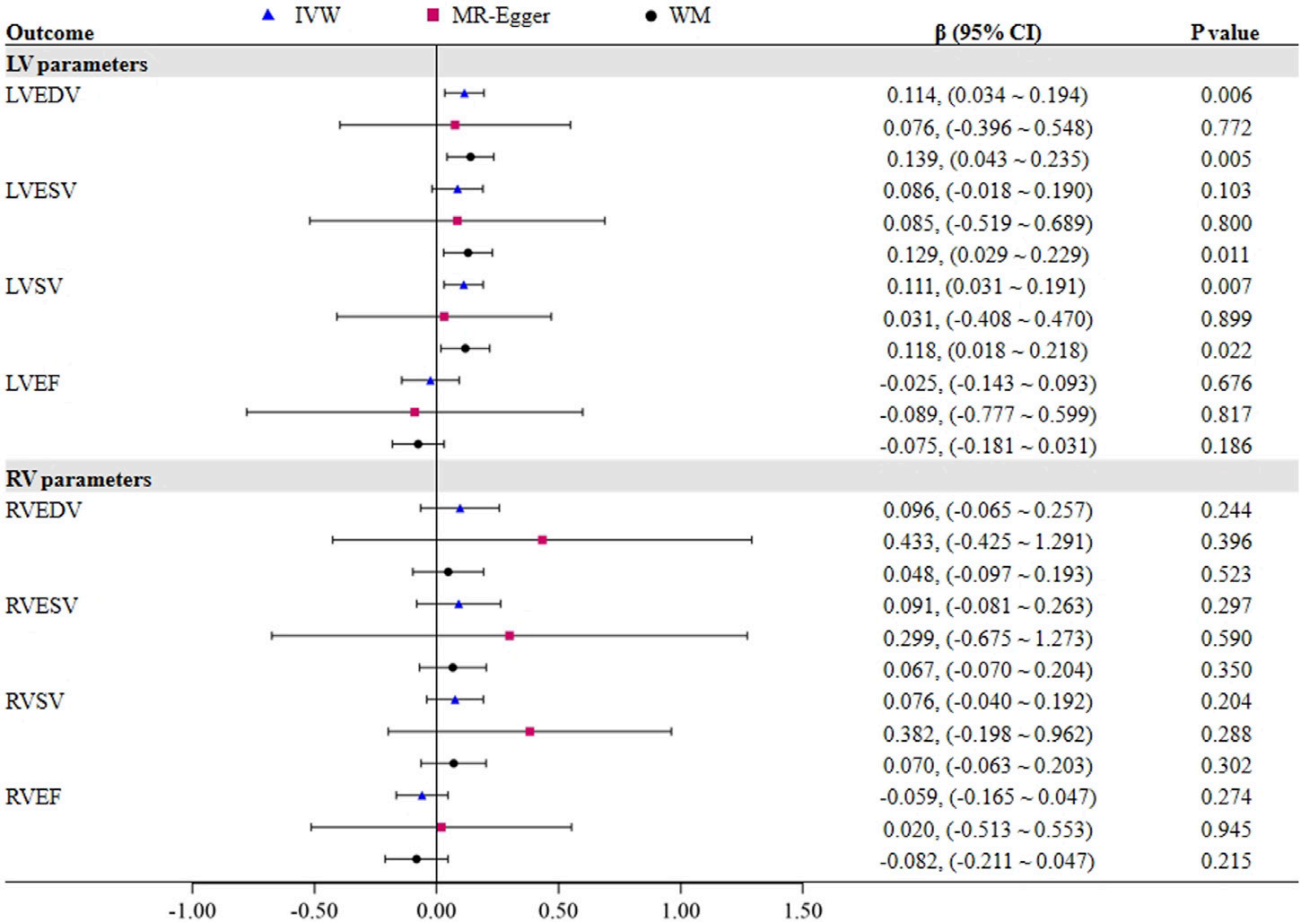
MR estimates for the causal effect of OSA on LV/RV parameters.

Figure 2 demonstrates a significant causal influence of OSA on LVEDV (*β* = 0.114, 95% CI = 0.034–0.194, *p* = 0.006) and LVSV (*β* = 0.111, 95% CI = 0.031–0.191, *p* = 0.007) in the IVW mode. The WM analysis yielded results that were consistent with the IVW approaches. Cochran Q test results in Table 1 revealed no evidence of heterogeneity on LVEDV (Cochran Q = 4.645, *p* = 0.326) and LVSV (Cochran Q = 3.617, *p* = 0.460), which is in line with the findings of MR-PRESSO. Furthermore, the MR-Egger intercept test for LVEDV (intercept = 0.004, *p* = 0.884) and LVSV (intercept = 0.007, *p* = 0.740) did not show any horizontal pleiotropic effects. Additionally, we performed a leave-one-out analysis; however, no SNP was found that significantly altered the IVW estimate. Nevertheless, the impact of OSA on LVESV and LVEF was not noted. Supplementary Tables S1–S4 presents the scatter plots and leave-one-out plots.

**TABLE 1:** The sensitivity results of OSA on LV/RV parameters.

Outcome	MR-PRESSO	Cochran Q test		MR-egger intercept	
	Global *p*-value	Q value	*p*-value	Intercept value	*p*-value
LV parameters					
LVEDV	0.423	4.645	0.326	0.004	0.884
LVESV	0.213	7.065	0.132	5.61E-05	0.999
LVSV	0.572	3.617	0.460	0.007	0.740
LVEF	0.196	7.142	0.129	0.006	0.865
RV parameters					
RVEDV	0.128	9.600	0.048	−0.031	0.490
RVESV	0.100	10.924	0.027	−0.019	0.699
RVSV	0.411	4.888	0.299	−0.029	0.369
RVEF	0.582	2.964	0.564	−0.007	0.786

We found no proof that OSA had a causal impact on any RV parameters (all *p* > 0.05). The Cochran Q test indicated that there seemed to be heterogeneity in OSA on RVEDV and RVESV, as shown in Table 1, despite the fact that MR-Egger intercept analysis revealed that none of the RV indicators exhibited horizontal pleiotropic effects. However, excluding the outlier rs10507084 separately did not significantly change our estimate of the causal effect (IVW: RVEDV, *β* = 0.050, 95% CI = −0.072–0.172, *p* = 0.425), and LVSV (IVW: RVESV, *β* = 0.054, 95% CI = −0.115–0.223, *p* = 0.530).

### 3.2 Causal effects of LV/RV on OSA

**FIGURE 3:**
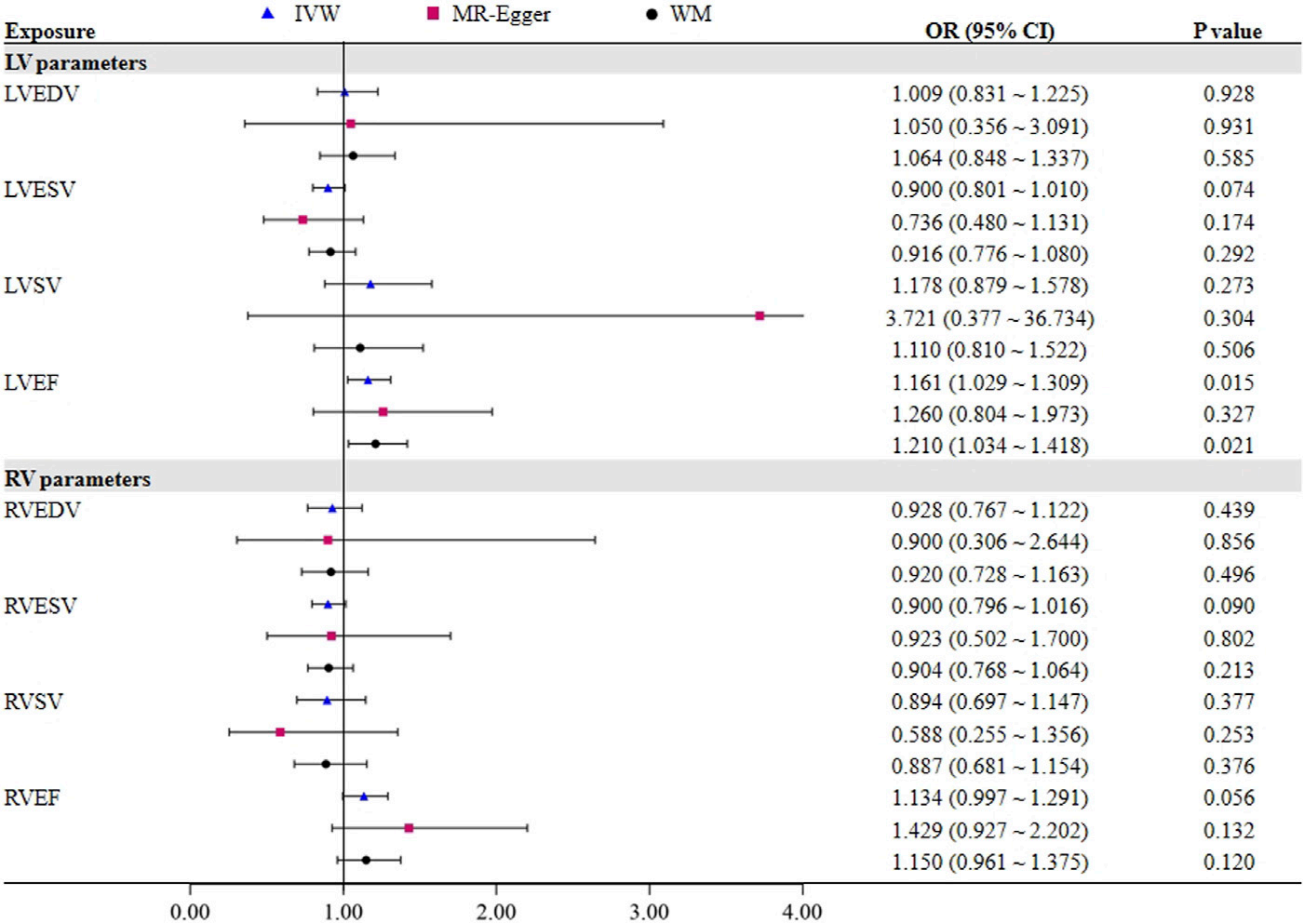
MR estimates for the causal effect of LV/RV parameters on OSA.

To further assess the causal effect of LV/RV on OSA, we conducted a reverse analysis. The information on IVs for LV/RV is provided in Supplementary Tables S2–S9. The F statistic value at each chosen IV was greater than 10, indicating that the chosen SNPs were adequately robust and that weak IVs were not likely to alter the causal estimate. Table 2 and Figure 3 indicate the genetic relationship between LV/RV parameters and OSA. The major MR analysis suggested that LVEF had a possible causal relationship with the risk of OSA (IVW: OR = 1.161, 95% CI = 1.029–1.309, *p* = 0.015). The relationship between LVEF and OSA risk is depicted in Supplementary Figure S5’s scatter plot. The Cochran Q statistic (Cochran Q = 23.334, *p* = 0.223), MR-Egger intercept test (intercept = −0.005, *p* = 0.714), and MR-PRESSO (Global *p* = 0.232) failed to identify any directional pleiotropy or heterogeneity.

**TABLE 2:** The sensitivity results of LV/RV parameters on OSA.

Exposure	MR-PRESSO	Cochran Q test		MR-egger intercept	
	Global *p*-value	Q value	*p*-value	Intercept value	*p*-value
LV parameters					
LVEDV	0.024	32.898	0.025	−0.002	0.943
LVESV	0.339	30.783	0.327	0.010	0.351
LVSV	0.080	13.641	0.058	−0.050	0.359
LVEF	0.232	23.334	0.223	−0.005	0.714
RV parameters					
RVEDV	0.871	2.620	0.855	0.002	0.958
RVESV	0.724	9.976	0.696	−0.002	0.933
RVSV	0.097	14.022	0.081	0.025	0.338
RVEF	0.599	11.088	0.603	−0.015	0.294

No one SNP significantly contributed to the link between LVEF and OSA, according to the leave-one-out sensitivity analysis (Supplementary Figure S6).

In addition, it did not appear that the other three LV parameters (LVEDV, LVESV, and LVSV) had a direct impact on the risk of OSA. In all analyses, no horizontal pleiotropic effects were discovered. Although heterogeneity was observed in LVEDV on OSA, the results were not significantly affected after deleting the outlier rs3918226 (IVW: OR = 1.050, 95% CI = 0.882–1.250, *p* = 0.581). In the reverse MR analysis, none of the methods produced statistically significant proof of a connection between the RV index and OSA risk (all *p* > 0.05).

## 4 Discussion

We studied the causality between OSA and LV/RV in this bidirectional MR research, and genetic variations that proxied the impact were discovered using publicly accessible large-scale GWAS data. Our data reveal that genetically predicted OSA is causally related to LVEDV and LVSV, whereas genetically determined LVEF has a causal impact on OSA risk. However, no proof of a causal relationship between any RV parameters and OSA was observed. Sensitivity analyses using several MR models showed that the conclusions were solid. This is the first bidirectional MR research that, to our knowledge, has looked at the causality between OSA and ventricular structure and function, minimizing biases in observational studies.

OSA is a ubiquitous sleep disorder which frequently arises in conjunction with cardiac dysfunction. Between 40% and 80% of people with hypertension, HF, CAD, pulmonary hypertension, AF, and stroke also have OSA (Yeghiazarians et al., 2021). The hallmarks of OSA are autonomic fluctuations, intermittent hypoxemia, and sleep disturbances, which trigger a range of pathophysiological mechanisms that may contribute to adverse cardiac structures and cardiovascular events. These mechanisms include sympathetic activation, oxidative stress, inflammation, endothelial dysfunction and thrombosis (Belaidi et al., 2009; Baguet et al., 2010; Bisogni et al., 2016). When negative intrathoracic pressure fluctuates widely, preload and afterload increase during obstructive breathing, which may lead to ventricular restructuring and diastolic dysfunction. All of these have a detrimental effect on the cardiovascular system, raising CVD morbidity and mortality.

In the last few decades, there has been a lot of observational evidence connecting OSA to the emergence of LV dysfunction. For instance, a prospective trial of 425 participants discovered that the risk of developing LV diastolic dysfunction was considerably enhanced in participants with moderate to severe OSA (Kidawara et al., 2022). These findings imply that OSA is a substantial and independent indicator of LV diastolic dysfunction progression, helping to improve disease prediction and prevention. A prospective multicenter cohort research with 1506 Hispanics/Latinos found similar results: severe sleep apnea was linked to subclinical signs of LV hypertrophy and diastolic dysfunction, which could exacerbate the HF burden under this cohort (Ogilvie et al., 2020). Selection bias, unmeasured confounding variables, and limited follow-up times may restrict these observational studies. This work used a two-way MR approach to determine the genetic link between OSA and LV, which may control for residual confounding factors, causality, and causal direction (Allman et al., 2018). Our findings suggest that early diagnosis, treatment, and follow-up of OSA patients may be necessary to prevent cardiac dysfunction and cardiovascular risk events. Previous studies support our findings that CPAP ventilation promotes LV function among OSA subjects compared with sham treatment (Shim et al., 2018; Kim et al., 2019).

Since most prior research on OSA and LVEF was done on subjects with CVD, it is hard to completely exclude reverse causal pathways (Hammerstingl et al., 2013; Wachter et al., 2013). For instance, a study with 252 stable HF patients indicated that sleep apnea was prevalent among HF patients and that the prevalence varied based on the LVEF, which was mostly connected to variations in cardiac function (Wang et al., 2022). According to their LVEF, the prevalence and severity of HF with reduced EF (HFrEF; defined as EF < 40%) and HF with mid-range EF (HFmrEF; defined as EF 40–49%) were significantly higher than those of HF with preserved EF (HFpEF; defined as EF ≥ 40%) (Savarese et al., 2022). As a result, to some extent, LVEF could forecast the incidence of sleep apnea. Comparable results were seen in a single-centre retrospective investigation that included 221 patients admitted for acute HF: sleep breathing problems were quite common in all three subgroups and were frequently more severe in HFrEF (Maltes et al., 2022). In contrast, another study with 101 Japanese patients with new-onset HF did not discover a link between sleep apnea and LVEF (Sekizuka et al., 2013). Various OSA diagnostic criteria, sample sizes, and research populations may have contributed to the inconsistent results of prior investigations. Our research demonstrates that an elevated risk of OSA is associated with genetically predicted LVEF. Therefore, for CVD patients with reduced LVEF, it is necessary to prevent OSA risk events.

The RV, which plays a vital role in various cardiac diseases, is coming under more and more scrutiny in research. According to previous research, RV structure and function are altered in people with OSA. As an illustration, a cross-sectional analysis compared patients without HF or chronic lung illness who had OSA and compared them to controls who did not (IbnHadj et al., 2022). Early in the disease’s progression, it was shown that OSA was independently correlated with structural variations in the RV, indicating that early diagnosis and therapy are crucial for avoiding detrimental consequences. According to a meta-analysis of 25 trials involving 1,503 OSA patients and 796 controls, individuals with OSA exhibited dilated RV, thickened RV walls, and altered RV function (Maripov et al., 2017). In 151 patients with hypertrophic obstructive cardiomyopathy, OSA was found in 55.6% of cases, and both its prevalence and severity were independently linked to decreased RVEF (Wang et al., 2020). All MR methods employed in this study, however, failed to detect any causal relationship between OSA and RV. Unknown confounding factors may still be at work in this disparity. Additionally, rather than being induced directly by OSA, RV dysfunction may be generated indirectly by the pulmonary vasoconstriction condition caused by intermittent hypoxia in OSA patients, resulting in increased RV burden in pulmonary arterial hypertension (Nokes et al., 2020).

The essential merit of this work is that the two-way MR analysis was utilized to eliminate two inherent flaws in typical observational studies: confounding factors and reverse causality. Moreover, our sensitivity analysis indicated no heterogeneity or pleiotropy, indicating that our conclusions are robust and reliable. Additionally, given that CMR is currently the gold standard for non-invasive clinical evaluation of heart function and allows to measure and evaluate the structure and function of the heart objectively, it was used to explore LV/RV parameters in contrast to the majority of studies that have examined cardiac function using echocardiography. Last but not least, the data we utilize comes from a large-scale GWAS, which has a substantial sample size to obtain statistically reliable power. Some limitations must be acknowledged in this investigation. First of all, while restricting the sample to individuals with European ancestry lessens demographic bias, it could make it more difficult to apply our findings to other populations. Second, various severity distributions and subtypes of OSA were not distinguished in this research. Furthermore, some CMR markers of cardiac function, like myocardial mass, were not sufficiently explored in this investigation. Lastly, while our findings merely show the overall association between OSA and LV/RV, the underlying mechanism merits more profound research.

## 5 Conclusion

In conclusion, we provided evidence for a causal association between OSA and ventricular structure, minimizing bias in observational studies and aiding clinical decision-making and primary prevention. Early diagnosis, treatment, and follow-up of patients with OSA may be necessary to prevent cardiac dysfunction and cardiovascular risk events. Therefore, the diagnosis and treatment of OSA should be further studied to provide more evidence for primary prevention. In addition, the direct mechanism between OSA and ventricular structure has not been confirmed, so future basic experimental research should focus on the specific pathophysiological mechanisms.

## Figures and Tables

**Table:** 

**AF**	Atrial fibrillation
**CAD**	Coronary artery disease
**CMR**	Cardiovascular magnetic resonance
**CPAP**	Continuous positive airway pressure
**CVD**	Cardiovascular disease
**GWAS**	Genome-wide association studies
**HF**	Heart failure
**HFmrEF**	Heart failure with mid-range ef
**HFpEF**	Heart failure with preserved ef
**HFrEF**	Heart failure with reduced ef
**ICD**	International classification of diseases
**IVs**	Instrumental variables
**IVW**	Inverse variance weighted
**LD**	Linkage disequilibrium
**LV**	Left ventricular
**LVEDV**	Left ventricular end-diastolic volume
**LVEF**	Left ventricular ejection fraction
**LVESV**	Left ventricular end-systolic volume
**LVSV**	Left ventricular stroke volume
**MR**	Mendelian randomization
**MR-PRESSO**	Mr pleiotropy residual sum and outlier
**OSA**	Obstructive sleep apnea
**RV**	Right ventricular
**RVEDV**	Right ventricular end-diastolic volume
**RVEF**	Right ventricular ejection fraction
**RVESV**	Right ventricular end-systolic volume
**RVSV**	Right ventricular stroke volume
**SNPs**	Single nucleotide polymorphisms
**WM**	Weighted median

## Data Availability

Publicly available datasets were analyzed in this study. This data can be found here: Data on OSA can be downloaded from https://www.finngen.fi/en. Data on LV/RV structure and function can be downloaded from https://www.ebi.ac.uk/gwas/.
