# Supplementary material for: The causality between obstructive sleep apnea and ventricular structure and function: a bidirectional Mendelian randomization study
Source: Front Genet. 2023 Oct 10;14:1266869. doi: 10.3389/fgene.2023.1266869 (PMC10597648; doi:10.3389/fgene.2023.1266869)
Supplement: Supplementary file 1 [file DataSheet1.PDF]

# Supplementary tables and figures

**Table S1** Information on instrumental variables of OSA.

| SNP | effect_allele | other_allele | eaf | beta | se | p value | $R^2$ | F |
|---|---|---|---|---|---|---|---|---|
| rs10928560 | T | C | 0.1949 | -0.0878 | 0.0158 | 2.80E-08 | 0.00241925 | 528.5614488 |
| rs4837016 | A | G | 0.4662 | -0.0706 | 0.0125 | 1.53E-08 | 0.002480791 | 542.0406036 |
| rs10507084 | T | C | 0.1793 | 0.1085 | 0.0163 | 2.80E-11 | 0.003464609 | 757.7471634 |
| rs9937053 | A | G | 0.4298 | 0.102 | 0.0125 | 4.32E-16 | 0.005099457 | 1117.138828 |
| rs142006783 | C | T | 0.03778 | 0.1783 | 0.0327 | 4.81E-08 | 0.002311368 | 504.9365896 |

Note: SNP, single nucleotide polymorphism; eaf, effect allele frequency; se, stantard error; F, F statistics; OSA, obstructive sleep apnea.

**Table S2** Information on instrumental variables of LVEDV.

| SNP | effect_allele | other_allele | eaf | beta | se | p value | $R^2$ | F |
|---|---|---|---|---|---|---|---|---|
| rs28579893 | A | G | 0.326721 | -0.036882 | 0.00620628 | 1.00E-09 | 0.000598454 | 21.58061198 |
| rs9429079 | T | C | 0.563921 | -0.0348721 | 0.00589983 | 5.30E-09 | 0.000598094 | 21.56761875 |
| rs7605066 | C | T | 0.429353 | 0.0331572 | 0.0059433 | 1.60E-08 | 0.000538726 | 19.42560234 |
| rs1873164 | G | A | 0.199952 | -0.0604022 | 0.00734999 | 1.20E-16 | 0.001167286 | 42.11698556 |
| rs73028849 | G | C | 0.658476 | 0.0395947 | 0.00621085 | 2.10E-10 | 0.000705124 | 25.42988283 |
| rs6777123 | A | C | 0.393323 | 0.0335026 | 0.00602949 | 1.80E-08 | 0.000535666 | 19.31520511 |
| rs28752924 | T | C | 0.55295 | 0.0347902 | 0.00610845 | 3.50E-08 | 0.000598392 | 21.57836307 |
| rs9275587 | T | C | 0.5249 | 0.0369598 | 0.0059508 | 8.90E-10 | 0.00068132 | 24.5708143 |
| rs2146324 | A | C | 0.261093 | 0.0386355 | 0.00677893 | 1.00E-08 | 0.000575954 | 20.76878147 |
| rs11153730 | T | C | 0.51257 | -0.0481809 | 0.00588735 | 3.70E-16 | 0.001159966 | 41.85256141 |
| rs3918226 | C | T | 0.920901 | 0.0584782 | 0.0110446 | 4.90E-08 | 0.000498198 | 17.96350487 |
| rs55679363 | A | T | 0.686166 | 0.041041 | 0.00635371 | 4.30E-11 | 0.000725429 | 26.16272323 |
| rs72840788 | G | A | 0.785239 | 0.0549298 | 0.00723155 | 1.50E-14 | 0.001017661 | 36.71286113 |
| rs7306710 | T | C | 0.479866 | 0.0390705 | 0.00596146 | 5.90E-11 | 0.000762014 | 27.4831782 |
| rs3184504 | T | C | 0.470291 | -0.0476702 | 0.00589672 | 3.30E-16 | 0.001132213 | 40.85005892 |

| | | | | | | | | |
|---|---|---|---|---|---|---|---|---|
| rs35828350 | G | A | 0.737748 | -0.038117 | 0.0066651 | 5.00E-09 | 0.000562204 | 20.27268314 |
| rs71385734 | T | G | 0.827608 | 0.0504053 | 0.00786999 | 2.10E-10 | 0.000724977 | 26.14639941 |
| rs2302455 | G | A | 0.884191 | 0.0591085 | 0.00933755 | 1.30E-10 | 0.000715514 | 25.80488101 |
| rs2231935 | C | G | 0.65653 | -0.0362847 | 0.00620152 | 8.20E-09 | 0.000593773 | 21.41169931 |
| rs12460541 | G | A | 0.653315 | -0.0397865 | 0.00616501 | 6.90E-11 | 0.000717066 | 25.86088663 |

Note: SNP, single nucleotide polymorphism; eaf, effect allele frequency; se, stantard error; F, F statistics; LVEDV, left ventricular end-diastolic volume.

**Table S3** Information on instrumental variables of LVESV.

| SNP | effect_allele | other_allele | eaf | beta | se | p value | $R^2$ | F |
|---|---|---|---|---|---|---|---|---|
| rs114300540 | C | T | 0.875439 | -0.0589669 | 0.00964533 | 3.20E-10 | 0.000758324 | 27.34996393 |
| rs1048302 | T | G | 0.326735 | -0.0604945 | 0.0063967 | 4.40E-22 | 0.001610065 | 58.11871632 |
| rs9429079 | T | C | 0.563921 | -0.0360857 | 0.00608496 | 4.30E-09 | 0.000640448 | 23.09588821 |
| rs2562845 | T | C | 0.803215 | 0.0770282 | 0.0075847 | 1.30E-23 | 0.001875656 | 67.72380955 |
| rs10167919 | C | T | 0.515291 | -0.0400483 | 0.00607674 | 1.60E-11 | 0.000801183 | 28.89699138 |
| rs11710541 | T | C | 0.658466 | 0.0583374 | 0.00639471 | 4.90E-20 | 0.001530705 | 55.24963991 |
| rs13092177 | G | T | 0.849986 | 0.0480731 | 0.00851759 | 1.30E-08 | 0.000589356 | 21.25233133 |
| rs1499813 | T | C | 0.590989 | 0.0351279 | 0.00616764 | 6.90E-09 | 0.000596553 | 21.51199288 |
| rs9274626 | T | C | 0.318688 | 0.039289 | 0.00665009 | 2.90E-09 | 0.000670322 | 24.17395123 |
| rs730506 | G | C | 0.799964 | 0.0531795 | 0.00762814 | 2.10E-12 | 0.000905101 | 32.64848928 |
| rs11153730 | T | C | 0.51257 | -0.0375259 | 0.00607781 | 6.80E-10 | 0.000703652 | 25.37675578 |
| rs34373805 | C | T | 0.838016 | 0.0554484 | 0.00828928 | 9.20E-12 | 0.000834704 | 30.10702571 |
| rs34866937 | G | A | 0.686563 | 0.0466226 | 0.00655078 | 8.70E-13 | 0.000935521 | 33.74681917 |
| rs1962104 | T | C | 0.448883 | -0.0370999 | 0.00612991 | 2.20E-09 | 0.000681008 | 24.55958536 |
| rs72840788 | G | A | 0.785239 | 0.0865583 | 0.00745607 | 8.10E-32 | 0.002526997 | 91.30115945 |

| | | | | | | | | |
|---|---|---|---|---|---|---|---|---|
| rs11604807 | T | C | 0.861802 | -0.0492542 | 0.00879674 | 3.50E-08 | 0.000577864 | 20.83768929 |
| rs113819537 | C | G | 0.749063 | -0.0395083 | 0.00698835 | 1.30E-08 | 0.000586799 | 21.16008359 |
| rs3184504 | T | C | 0.470291 | -0.0387589 | 0.00608541 | 1.10E-10 | 0.000748474 | 26.99447049 |
| rs116904997 | G | A | 0.977699 | -0.112497 | 0.020597 | 3.40E-08 | 0.000551876 | 19.90003596 |
| rs35630683 | T | C | 0.737566 | -0.0486324 | 0.00687127 | 5.70E-13 | 0.000915593 | 33.02729001 |
| rs67918525 | A | G | 0.670882 | 0.0362356 | 0.0067551 | 1.40E-08 | 0.000579827 | 20.9085206 |
| rs71385734 | T | G | 0.827608 | 0.0531472 | 0.00812096 | 4.50E-11 | 0.000805995 | 29.070699 |
| rs2302455 | G | A | 0.884191 | 0.0634669 | 0.00963271 | 8.60E-11 | 0.000824922 | 29.75391431 |
| rs12452367 | T | C | 0.714885 | 0.0473424 | 0.00676288 | 5.60E-13 | 0.000913665 | 32.95766995 |
| rs9897002 | A | G | 0.570512 | 0.0326517 | 0.0061377 | 4.00E-08 | 0.000522465 | 18.83896778 |
| rs10871753 | G | T | 0.489283 | 0.0329825 | 0.00610155 | 3.00E-08 | 0.000543673 | 19.60408102 |
| rs62130043 | A | G | 0.338621 | 0.0361349 | 0.00646336 | 1.30E-08 | 0.000584855 | 21.08991379 |
| rs10421891 | A | G | 0.645254 | -0.0450999 | 0.00632234 | 6.70E-13 | 0.000931171 | 33.5897436 |
| rs2267038 | G | C | 0.194083 | -0.055643 | 0.00771188 | 1.30E-13 | 0.000968565 | 34.93996465 |

Note: SNP, single nucleotide polymorphism; eaf, effect allele frequency; se, stantard error; F, F statistics; LVESV, left ventricular end-systolic volume.

**Table S4** Information on instrumental variables of LVSV.

| SNP | effect_allele | other_allele | eaf | beta | se | p value | $R^2$ | F |
|---|---|---|---|---|---|---|---|---|
| rs7573293 | C | T | 0.274714 | -0.0492144 | 0.00705061 | 2.00E-12 | 0.000965171 | 34.81741909 |
| rs4868241 | C | T | 0.397549 | -0.0392855 | 0.00646233 | 1.30E-09 | 0.000739277 | 26.66250039 |
| rs28752900 | T | A | 0.559885 | 0.0381465 | 0.00658835 | 6.80E-09 | 0.000717141 | 25.8635828 |
| rs2146324 | A | C | 0.261093 | 0.0411111 | 0.00724306 | 1.10E-08 | 0.000652129 | 23.51739639 |
| rs72967533 | T | C | 0.520957 | -0.051094 | 0.00631957 | 1.40E-15 | 0.001303005 | 47.02027536 |
| rs10400419 | T | C | 0.447903 | 0.0401541 | 0.00647567 | 5.40E-10 | 0.000797424 | 28.76128816 |
| rs11065979 | C | T | 0.571948 | 0.0482938 | 0.00638164 | 3.90E-14 | 0.001141999 | 41.20356652 |
| rs422068 | T | C | 0.641519 | 0.0394527 | 0.00653726 | 1.40E-09 | 0.000715911 | 25.81920592 |
| rs143384 | A | G | 0.588391 | -0.0348726 | 0.00638051 | 3.60E-08 | 0.000589046 | 21.24115668 |

Note: SNP, single nucleotide polymorphism; eaf, effect allele frequency; se, stantard error; F, F statistics; LVSV, left ventricular stroke volume.

**Table S5** Information on instrumental variables of LVEF.

| SNP | effect_allele | other_allele | eaf | beta | se | p value | $R^2$ | F |
|---|---|---|---|---|---|---|---|---|
| rs2503715 | A | G | 0.127509 | -0.0584299 | 0.010793 | 4.00E-08 | 0.00075963 | 27.39711531 |
| rs1739837 | C | T | 0.410672 | 0.0707883 | 0.00695475 | 2.90E-25 | 0.002425522 | 87.62590817 |
| rs10925197 | C | G | 0.462051 | 0.0420377 | 0.00692711 | 6.90E-10 | 0.000878494 | 31.68789078 |
| rs2562845 | T | C | 0.803215 | -0.0792249 | 0.00863815 | 5.70E-20 | 0.001984162 | 71.64938962 |
| rs11710541 | T | C | 0.658466 | -0.0650511 | 0.00728417 | 9.10E-20 | 0.001903297 | 68.72372568 |
| rs56099248 | C | T | 0.805286 | -0.0607354 | 0.00873548 | 5.10E-12 | 0.001156808 | 41.73847075 |
| rs35999985 | A | G | 0.298992 | 0.0479992 | 0.00756877 | 3.80E-10 | 0.000965785 | 34.83957961 |
| rs9274626 | T | C | 0.318688 | -0.0417383 | 0.00756899 | 2.70E-08 | 0.000756504 | 27.28429012 |
| rs3176326 | G | A | 0.800287 | -0.0776886 | 0.00871931 | 1.80E-19 | 0.001929286 | 69.66395123 |
| rs3807309 | G | A | 0.886479 | -0.0834801 | 0.0109335 | 4.10E-15 | 0.001402622 | 50.62008701 |
| rs57655799 | A | C | 0.44789 | -0.0379582 | 0.00695732 | 1.30E-08 | 0.000712587 | 25.69925357 |
| rs4073554 | T | C | 0.480844 | 0.0428024 | 0.0069512 | 1.10E-09 | 0.000914678 | 32.99426593 |
| rs72840788 | G | A | 0.785239 | -0.0996914 | 0.00848638 | 4.00E-32 | 0.00335199 | 121.2086439 |
| rs721067 | T | A | 0.917806 | 0.0696667 | 0.0126693 | 3.60E-08 | 0.00073227 | 26.40963213 |
| rs113819537 | C | G | 0.749063 | 0.044906 | 0.0079573 | 1.70E-08 | 0.000758092 | 27.34159706 |

| | | | | | | | | |
|---|---|---|---|---|---|---|---|---|
| rs8023658 | G | T | 0.507123 | -0.0463516 | 0.00705314 | 4.60E-11 | 0.001074017 | 38.74812913 |
| rs5029142 | T | A | 0.623384 | -0.0430463 | 0.00718653 | 3.90E-10 | 0.000870074 | 31.38389442 |
| rs12452367 | T | C | 0.714885 | -0.0550497 | 0.00769448 | 1.30E-13 | 0.001235367 | 44.57647643 |
| rs2047273 | T | C | 0.677702 | -0.0417641 | 0.00752275 | 3.10E-08 | 0.000761961 | 27.4812448 |
| rs10871753 | G | T | 0.489283 | -0.0430639 | 0.00694036 | 6.10E-10 | 0.000926824 | 33.43278735 |
| rs2070458 | A | T | 0.200222 | 0.062928 | 0.00869212 | 2.50E-13 | 0.001268233 | 45.76389395 |

Note: SNP, single nucleotide polymorphism; eaf, effect allele frequency; se, stantard error; F, F statistics; LVEF, left ventricular ejection fraction.

**Table S6** Information on instrumental variables of RVEDV.

| SNP | effect_allele | other_allele | eaf | beta | se | p value | $R^2$ | F |
|---|---|---|---|---|---|---|---|---|
| rs11083473 | A | G | 0.447465 | -0.044675 | 0.0081236 | 3.80E-08 | 0.000986911 | 29.14658676 |
| rs2042995 | T | C | 0.7773 | 0.0714919 | 0.00967615 | 1.50E-13 | 0.001769508 | 52.30011292 |
| rs2066332 | A | G | 0.627788 | 0.0464542 | 0.0084637 | 4.10E-08 | 0.001008517 | 29.78533307 |
| rs4766578 | T | A | 0.493179 | -0.0623497 | 0.00806417 | 1.10E-14 | 0.001943381 | 57.44915289 |
| rs61400540 | G | A | 0.676707 | -0.0492757 | 0.00866109 | 1.30E-08 | 0.001062411 | 31.37871212 |
| rs752650 | T | C | 0.650236 | -0.0481292 | 0.00848914 | 1.40E-08 | 0.001053643 | 31.11946562 |
| rs754020 | T | C | 0.445162 | -0.0492836 | 0.00819566 | 1.80E-09 | 0.001199828 | 35.44226083 |
| rs76774446 | C | A | 0.863027 | -0.0665791 | 0.0117581 | 1.50E-08 | 0.001048009 | 30.9529089 |

Note: SNP, single nucleotide polymorphism; eaf, effect allele frequency; se, stantard error; F, F statistics; RVEDV, reft ventricular end-diastolic volume.

**Table S7** Information on instrumental variables of RVESV.

| SNP | effect_allele | other_allele | eaf | beta | se | p value | $R^2$ | F |
|---|---|---|---|---|---|---|---|---|
| rs12126782 | T | G | 0.638589 | -0.0613953 | 0.00847764 | 4.40E-13 | 0.001739895 | 51.42333415 |
| rs2042995 | T | C | 0.777358 | 0.0837652 | 0.00965114 | 4.00E-18 | 0.002428765 | 71.83273521 |
| rs9856926 | C | A | 0.565795 | -0.0649495 | 0.00813176 | 1.40E-15 | 0.002072696 | 61.27982821 |
| rs2276773 | A | G | 0.495985 | -0.0596695 | 0.00808562 | 1.60E-13 | 0.00178011 | 52.614019 |
| rs55754224 | C | T | 0.744299 | 0.056904 | 0.00923656 | 7.20E-10 | 0.001232524 | 36.40926878 |
| rs6813098 | G | A | 0.153408 | 0.0640285 | 0.0111497 | 9.30E-09 | 0.001064875 | 31.45157762 |
| rs72801474 | G | A | 0.904773 | 0.0759279 | 0.0137857 | 3.60E-08 | 0.000993419 | 29.33898313 |
| rs11784619 | G | A | 0.941727 | 0.0952555 | 0.0174055 | 4.40E-08 | 0.00099587 | 29.41142943 |
| rs72840788 | G | A | 0.782241 | 0.0834663 | 0.00986797 | 2.70E-17 | 0.002373389 | 70.19107319 |
| rs4766578 | T | A | 0.493177 | -0.0623815 | 0.00804511 | 8.90E-15 | 0.001945363 | 57.50787698 |
| rs61400540 | G | A | 0.676721 | -0.0493641 | 0.00863689 | 1.10E-08 | 0.001066202 | 31.49080643 |
| rs17608766 | T | C | 0.852244 | -0.0780404 | 0.0113473 | 6.10E-12 | 0.001533833 | 45.3237247 |
| rs503274 | C | T | 0.29944 | -0.0524707 | 0.00883245 | 2.80E-09 | 0.001155098 | 34.11942508 |
| rs56113315 | C | T | 0.514491 | -0.048752 | 0.00806552 | 1.50E-09 | 0.001187381 | 35.07412254 |
| rs10402263 | G | C | 0.65101 | -0.0501352 | 0.00845115 | 3.00E-09 | 0.001142132 | 33.7359812 |

Note: SNP, single nucleotide polymorphism; eaf, effect allele frequency; se, stantard error; F, F statistics; RVESV, reft ventricular end-systolic volume.

**Table S8** Information on instrumental variables of RVSV.

| SNP | effect_allele | other_allele | eaf | beta | se | p value | $R^2$ | F |
|---|---|---|---|---|---|---|---|---|
| rs11065979 | C | T | 0.562996 | 0.0426429 | 0.00824186 | 2.30E-07 | 0.000894776 | 26.42310499 |
| rs111743217 | G | A | 0.908105 | -0.0793287 | 0.0154088 | 2.60E-07 | 0.001050313 | 31.02100784 |
| rs365990 | A | G | 0.631495 | 0.0450722 | 0.00846084 | 1.00E-07 | 0.000945498 | 27.92238614 |
| rs3918226 | C | T | 0.919245 | 0.0817861 | 0.015263 | 8.40E-08 | 0.000993092 | 29.32932717 |
| rs3951016 | T | A | 0.533208 | 0.0489323 | 0.00825373 | 3.10E-09 | 0.001191904 | 35.20790321 |
| rs4295863 | C | A | 0.552893 | -0.0456261 | 0.00824011 | 3.10E-08 | 0.001029222 | 30.39746504 |
| rs4590694 | C | T | 0.824271 | 0.0561173 | 0.0110507 | 3.80E-07 | 0.000912299 | 26.94103494 |
| rs4811601 | C | T | 0.538619 | -0.0462085 | 0.00831126 | 2.70E-08 | 0.001061244 | 31.34419706 |
| rs73810962 | G | A | 0.879223 | 0.066769 | 0.0125916 | 1.10E-07 | 0.00094681 | 27.96116784 |
| rs78427222 | T | G | 0.956101 | -0.103723 | 0.0200829 | 2.40E-07 | 0.000903106 | 26.66931277 |

Note: SNP, single nucleotide polymorphism; eaf, effect allele frequency; se, stantard error; F, F statistics; RVSV, reft ventricular stroke volume.

**Table S9** Information on instrumental variables of RVEF.

| SNP | effect_allele | other_allele | eaf | beta | se | p value | $R^2$ | F |
|---|---|---|---|---|---|---|---|---|
| rs9442216 | T | C | 0.330853 | 0.0472197 | 0.00862712 | 4.40E-08 | 0.000987263 | 29.1570062 |
| rs78529941 | G | A | 0.619079 | 0.0527936 | 0.00841206 | 3.50E-10 | 0.001314539 | 38.83521475 |
| rs10930846 | A | G | 0.73684 | -0.0514181 | 0.00933576 | 3.60E-08 | 0.00102531 | 30.28178911 |
| rs55834511 | G | C | 0.785292 | 0.0721642 | 0.00999901 | 5.30E-13 | 0.001756115 | 51.90357108 |
| rs2276773 | A | G | 0.495952 | 0.0479352 | 0.0082087 | 5.20E-09 | 0.001148816 | 33.93366247 |
| rs6876106 | G | A | 0.709948 | -0.0539866 | 0.00901641 | 2.10E-09 | 0.00120034 | 35.45739846 |
| rs2572376 | C | A | 0.55236 | 0.0451076 | 0.00812649 | 2.80E-08 | 0.001006191 | 29.71656778 |
| rs11786896 | C | T | 0.949567 | -0.110708 | 0.0188386 | 4.20E-09 | 0.001173893 | 34.67523683 |
| rs2789750 | C | G | 0.67633 | 0.0572778 | 0.00882096 | 8.40E-11 | 0.001436361 | 42.43936766 |
| rs12006440 | C | T | 0.966958 | 0.138133 | 0.0231406 | 2.40E-09 | 0.001219267 | 36.01716826 |
| rs11007712 | A | G | 0.74599 | -0.0555189 | 0.00964084 | 8.50E-09 | 0.001168142 | 34.50515916 |
| rs72840788 | G | A | 0.782279 | -0.0886398 | 0.0100042 | 8.00E-19 | 0.002676391 | 79.17613947 |
| rs79884713 | G | A | 0.975261 | -0.156324 | 0.0265037 | 3.70E-09 | 0.001179191 | 34.8319373 |
| rs501740 | G | T | 0.260965 | 0.0609755 | 0.00931294 | 5.90E-11 | 0.001434128 | 42.37329025 |

Note: SNP, single nucleotide polymorphism; eaf, effect allele frequency; se, stantard error; F, F statistics; RVEF, reft ventricular ejection fraction.

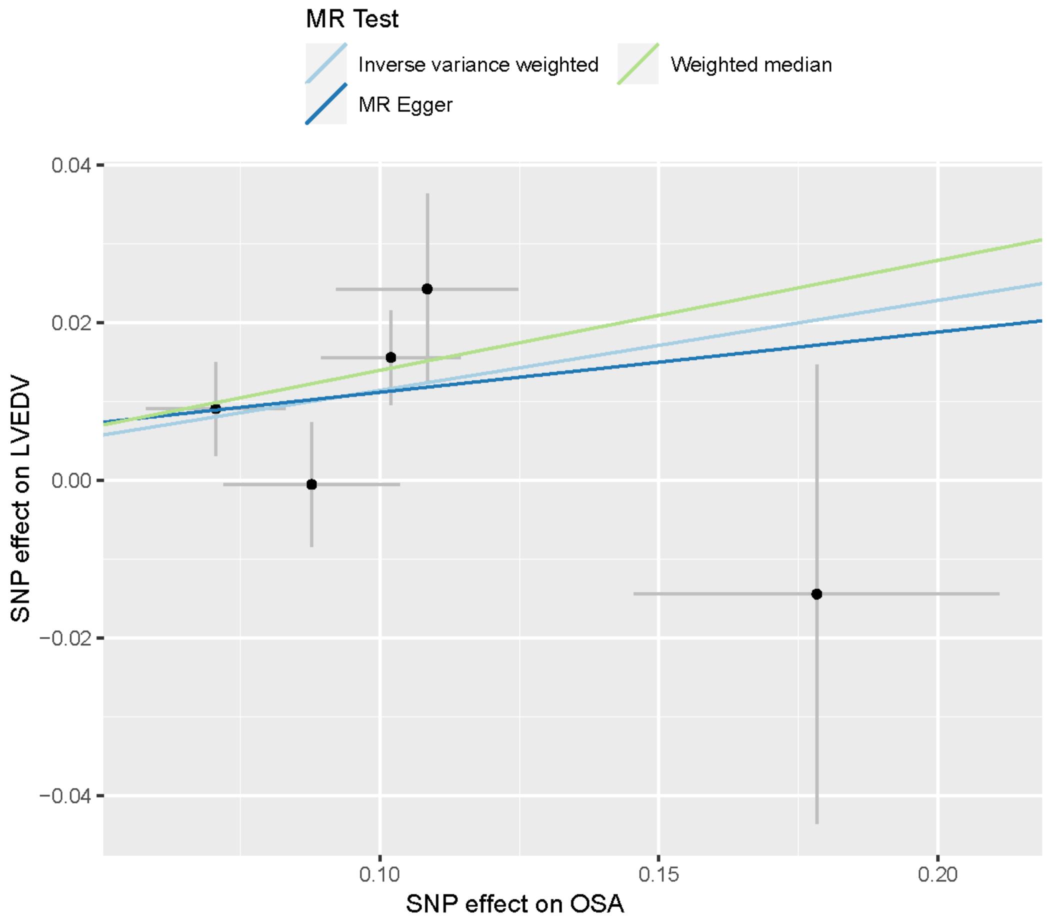


**Figure S1** Scatter plot for OSA on LVEDV.

Note: OSA, obstructive sleep apnea; LVEDV, left ventricular end-diastolic volume.

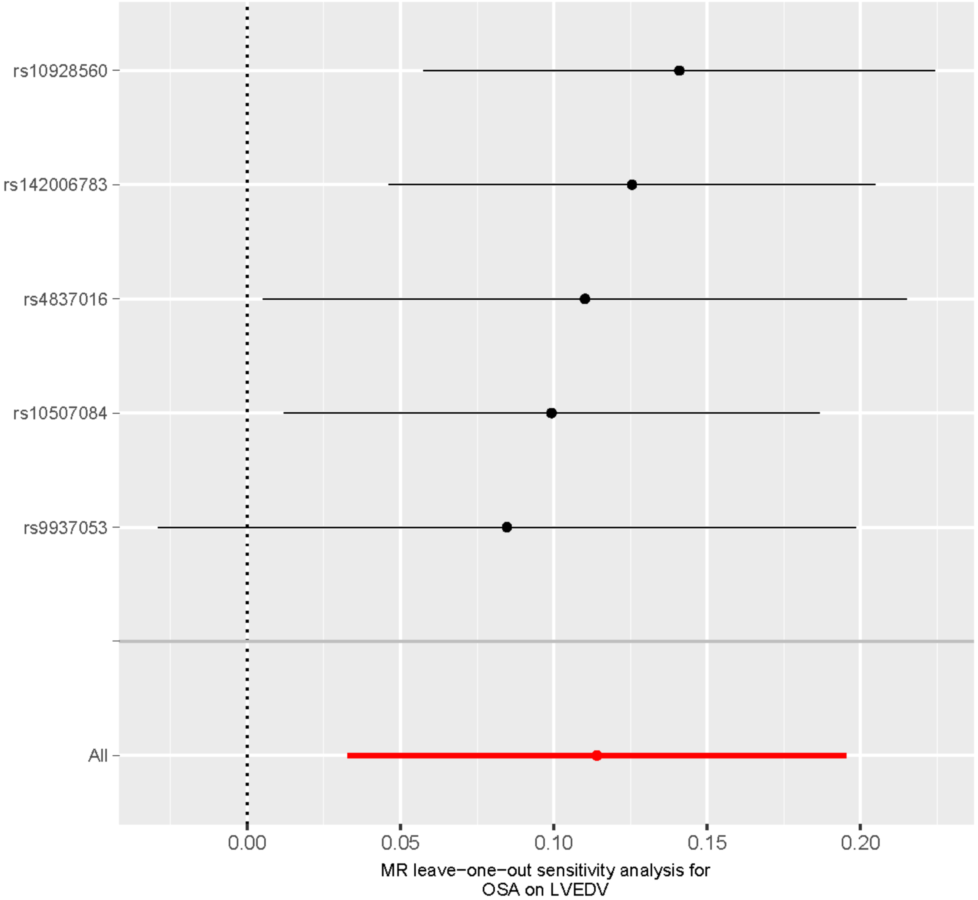


**Figure S2** Leave-one-out analysis for OSA on LVEDV.

Note: OSA, obstructive sleep apnea; LVEDV, left ventricular end-diastolic volume.

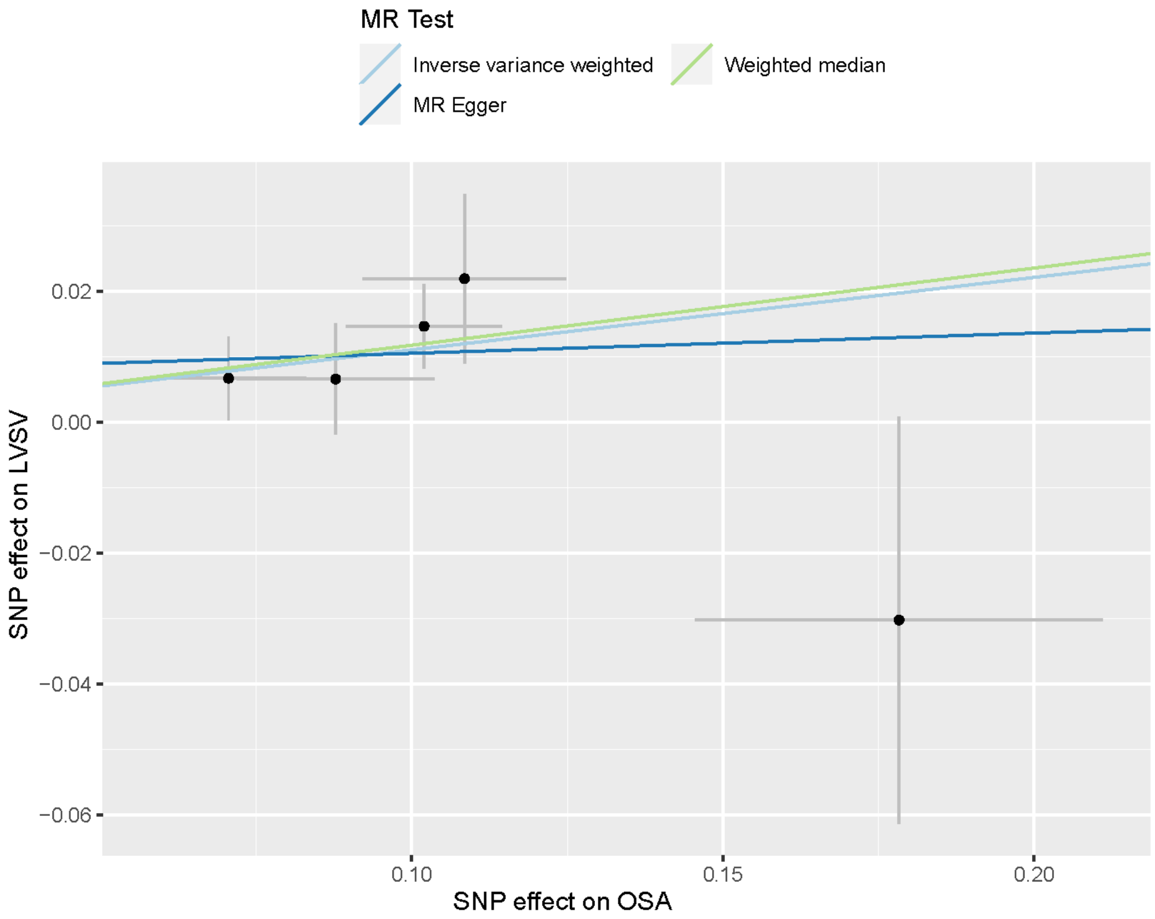


**Figure S3** Scatter plot for OSA on LVSV.

Note: OSA, obstructive sleep apnea; LVSV, left ventricular stroke volume.

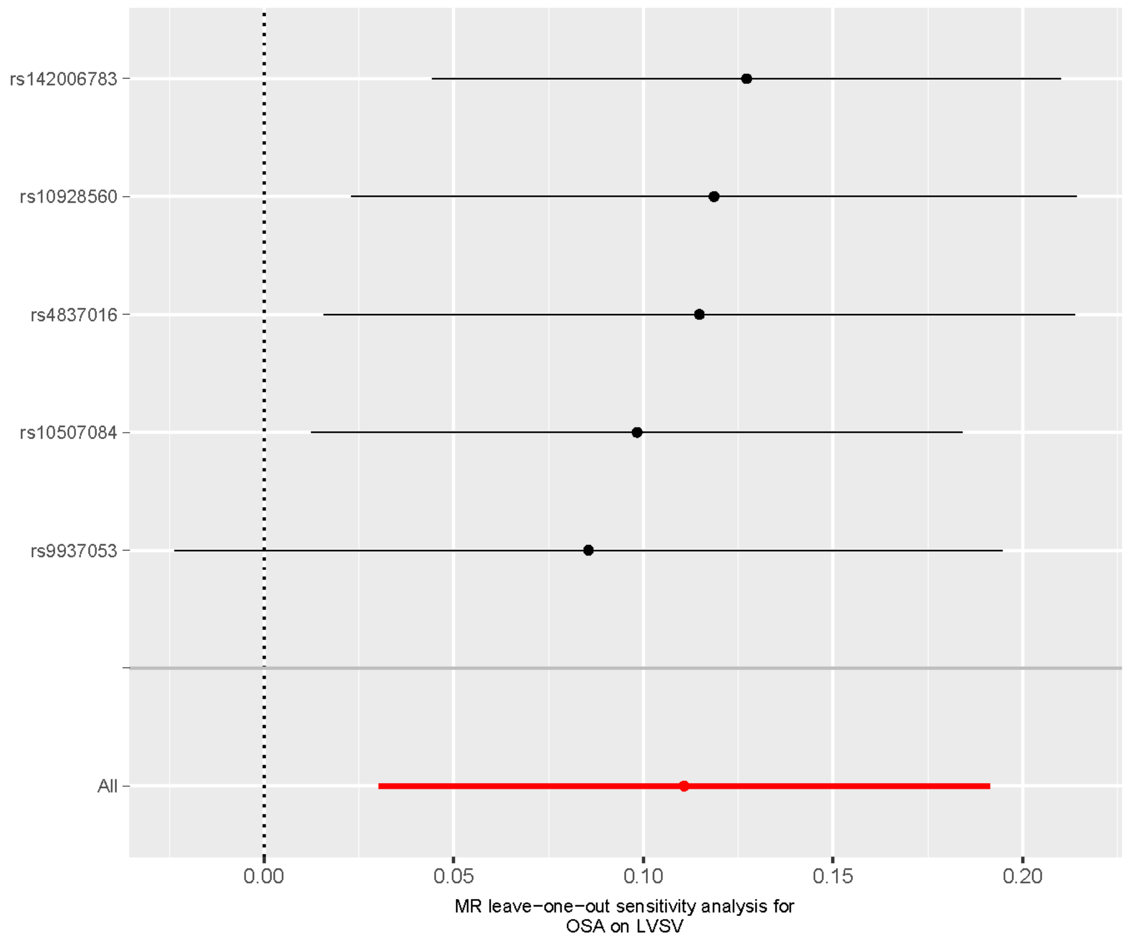


**Figure S4** Leave-one-out analysis for OSA on LVSV.

Note: OSA, obstructive sleep apnea; LVSV, left ventricular stroke volume.

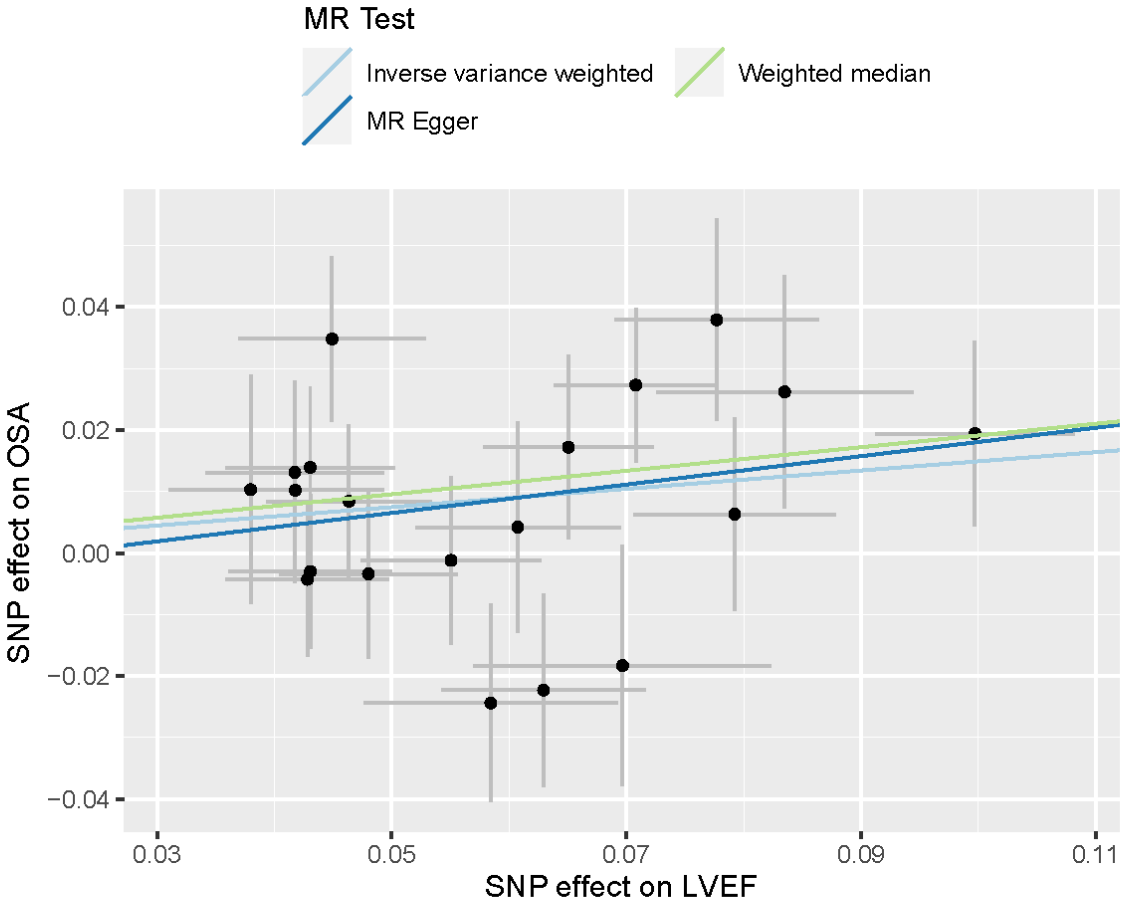


**Figure S5** Scatter plot for LVEF on OSA.

Note: LVEF, left ventricular ejection fraction; OSA, obstructive sleep apnea.

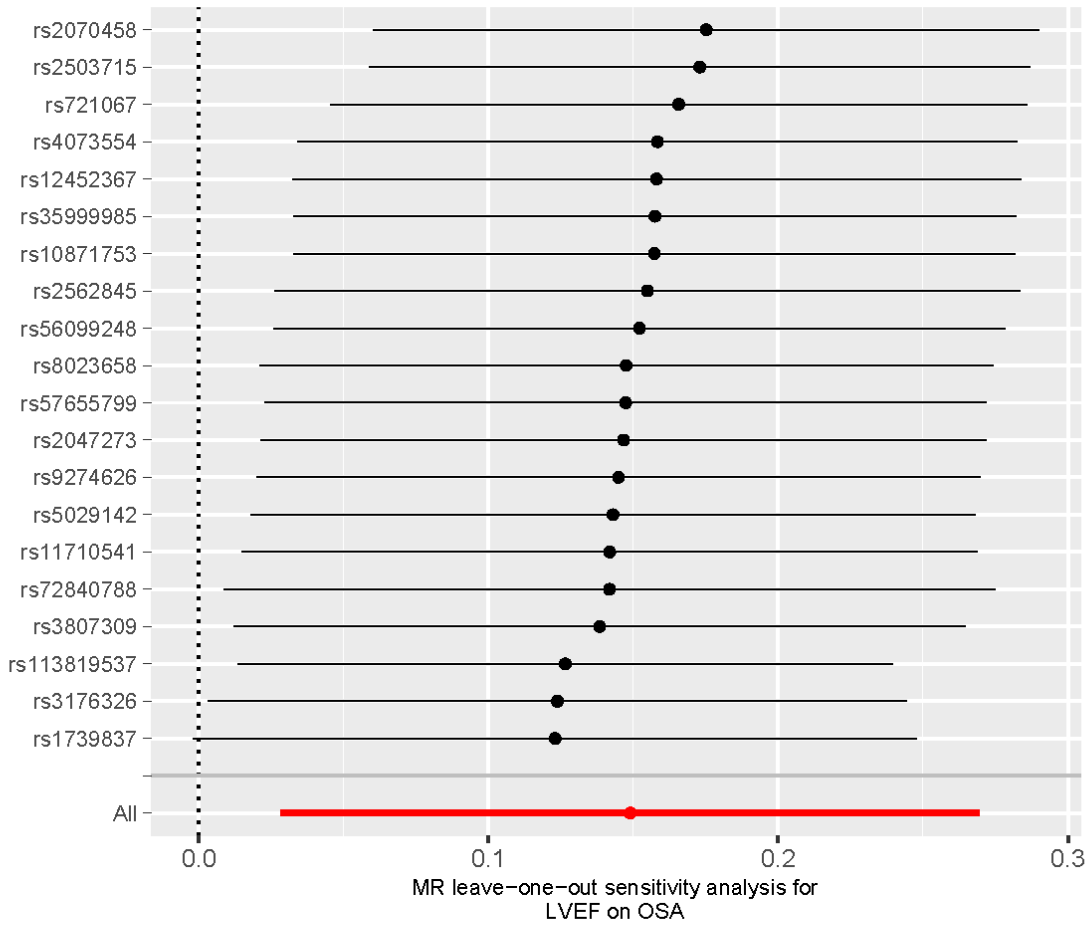


**Figure S6** Leave-one-out analysis for LVEF on OSA.

Note: LVEF, left ventricular ejection fraction; OSA, obstructive sleep apnea.
